# Realizing the potential of embedded implementation research: Lessons from Pakistan

**DOI:** 10.7189/jogh.10.020104

**Published:** 2020-12

**Authors:** Kumanan Rasanathan, Nhan Tran, Hope L Johnson, Assad Hafeez, Stefan Peterson, Abdul Ghaffar

**Affiliations:** 1 Health Section, UNICEF, New York, New York, USA; 2 Alliance for Health Policy and Systems Research, Geneva, Switzerland; 3 Gavi, the Vaccine Alliance, Geneva, Switzerland; 4 Ministry of National Health Services, Regulation and Coordination, Islamabad, Pakistan

At the inception meeting for the project in Pakistan that this supplement draws from, a provincial immunization manager complained about researchers. He was scathing in his assessment that researchers were not helpful to him, that their research was irrelevant to the problems he faced in his work, and moreover, that researchers did not engage with him or share their research. His contention was that their only concern was in publishing papers.

These questions about the usefulness of research and the behaviour of researchers may rarely be expressed so bluntly, but they reflect common concerns for policy-makers and programme managers [1]. The incentives of research and practice are often misaligned, and there is a disproportionate focus on research that addresses the “what” rather than the “how”, or on problems rather than solutions [2].

A nascent field, ‘embedded implementation research’ (the integration of research methods and approaches within existing health programme implementation and policymaking cycles in order to improve service delivery and overcome bottlenecks), represents an attempt to address these issues. If implementation research itself aims to squarely focus on the “how” of implementation [3], ‘embedded’ implementation research aims to supplement this focus by prioritizing the involvement and perspective of implementers in the research process itself [4,5]. This occurs through the positioning of research within the context of actual health programmes to solve challenges to service delivery, meaningful engagement and leadership by practitioners and decision-makers to ensure the relevance and application of research studies, and the alignment of research activities with implementation, funding and policy cycles, with continuing engagement. The studies in this supplement illustrate the purpose and potential of embedded implementation research while also highlighting some of the challenges.

This project arose out of an ongoing collaboration between UNICEF, the Alliance for Health Policy and Systems Research and Gavi, the Vaccine Alliance. The aim of this collaboration has been to provide local decision-makers with the key information they need while also building local capacity for implementation research, both of which are important to improve immunization service delivery and outcomes. Starting in 2014, UNICEF, the Alliance and Gavi have supported multiple research projects in several different countries. The project covered by this supplement in Pakistan resulted from the opportunity to use unspent Gavi funds for this model of embedded implementation research – which involved ensuring country leadership through the Ministry of National Health Services, Regulation and Coordination and the Health Services Academy in Pakistan, sensitizing stakeholders, developing a priority agenda of issues, and then issuing an open call for proposals from local teams that had to include both implementers and researchers. UNICEF and the Alliance facilitated technical support and training to develop protocols and execute studies over a short timeline of 1 year. A full description of the process is reported elsewhere [6].

This experience in Pakistan reinforces several lessons that have been highlighted by the broader experience of this collaboration. First, there is a growing demand and appreciation for this type of work, with implementers and policymakers keen to be involved in research when they consider it relevant to challenges they face. This is shown by the high number of submissions to the call (despite the short period) which mirrors the experience the collaboration has had in its other calls. Discussions with policy-makers and implementers have also revealed their satisfaction in being able to drive research rather than being passive consumers of end products.

Second, there is significant capacity in Pakistan to undertake this type of work, and studies can be undertaken in a short time period. When we embarked on this collaboration, we encountered skepticism about the timeframe, and whether there was sufficient capacity in low- and middle-income countries to apply for and undertake embedded implementation research. As with our previous calls, the Pakistan experience shows this is not the case, and that as in other settings, embedded implementation research can help to overcome implementation barriers through responding to the needs of decision-makers and being integrated into programme delivery. Embedded implementation research studies can also be undertaken in a relatively short period (in contrast to the usual lengthy research cycle) and at low cost to produce meaningful results for implementation in low- and middle-income country settings, where the capacity challenges in most settings are overrated. National institutional capacity is crucial but can also be built through these types of activities.

Third, despite these promising results, there are several areas for development. As development partner agencies, we need to continue to sensitize staff and build our own capacity to support embedded implementation research in countries. Senior leadership both within countries and within agencies also need further explanation and convincing around this approach. The involvement of implementers in this work is key, but turnover in programme managers is difficult to manage, as in many other activities, and can create challenges in implementing findings. There also remain challenges in fully aligning policy and funding cycles with studies, which is essential to maximizing the impact of the work. Within countries, implementers and researchers often still aspire to more traditional forms of research when undertaking embedded implementation research and sometimes need to be encouraged to narrow the scope of their proposals. These aspirations are linked to the incentive structures for academic researchers. And the ethical approval process is currently not well adapted to the needs of embedded implementation research. Ethics committees often have limited understanding of this type of research and onerous processes sometimes mitigate against the alignment with planning and implementation cycles aimed for by embedded implementation research.

The experience in Pakistan also points to some useful future directions for embedded implementation research as a field. A pressing need is to follow up existing studies to evaluate medium and long-term impact. Methodologies and the overall approach can also be refined based on existing experiences. As development partners, we can harmonize our approach and better coordinate our support in countries, using a shared language so as not to confuse on what is already a concept that is difficult to understand. Together, the priority is to build the capacity for embedded implementation research in national institutions and move this type of work to being “business as usual” rather than project-based, with dedicated national budget lines. And it is important to further clarify how embedded implementation research is complementary to, and not a substitute for, impact evaluation.

Beyond these steps, embedded implementation research successfully undertaken challenges the power relationships within the practice of health research itself. While researchers are generally keen to have greater use of their work by implementers, through this collaboration we have seen instances of discomfort by researchers in having implementers lead research or control research resources. Our aim however is exactly to subvert hierarchies in the execution of health research and production of health knowledge, and to facilitate genuine partnerships between researchers and implementers in the co-creation of solutions to health service delivery problems. This is a contribution to the broader mission of reorienting research to be more responsive to the realities and concerns of health system workers, to their planning and implementation cycles, and to how research knowledge is most easily consumed and applied.

We believe embedded implementation research has an important role in improving health service delivery, including for immunization, towards achievement of the health-related Sustainable Development Goals. While there is increasing understanding of its value, and already some demonstrable improvements in service delivery from this type of work, its full potential and impact remains to be realized, scrutinized and understood. The challenges noted above are not trivial, but they are mostly surmountable. The overarching conclusion from the experiences in Pakistan highlighted in this supplement is of the need to continue to foster the field of embedded implementation research, building capacity and overcoming these challenges.
